# Virtual methylome dissection facilitated by single-cell analyses

**DOI:** 10.1186/s13072-019-0310-9

**Published:** 2019-11-11

**Authors:** Liduo Yin, Yanting Luo, Xiguang Xu, Shiyu Wen, Xiaowei Wu, Xuemei Lu, Hehuang Xie

**Affiliations:** 1 State Key Laboratory of Genetic Resources and Evolution, Kunming Institute of Zoology, Chinese Academy of Sciences, Kunming, 650223 China; 2 Kunming College of Life Science, University of Chinese Academy of Sciences, Beijing, 100101 China; 3 Center for Excellence in Animal Evolution and Genetics, Chinese Academy of Sciences, Kunming, 650223 China; 4 Key Laboratory of Genomic and Precision Medicine, Beijing Institute of Genomics, Chinese Academy of Sciences, Beijing, 100101 China; 5 Epigenomics and Computational Biology Lab, Fralin Life Sciences Institute at Virginia Tech, Virginia Tech, Blacksburg, VA 24061 USA; 6 Department of Biological Sciences, Virginia Tech, Blacksburg, VA 24061 USA; 7 Department of Statistics, Virginia Tech, Blacksburg, VA 24061 USA; 8 Department of Biomedical Sciences and Pathobiology, Virginia-Maryland College of Veterinary Medicine, Virginia Tech, Blacksburg, VA 24061 USA; 9 School of Future Technology, University of Chinese Academy of Sciences, Beijing, 100101 China

**Keywords:** DNA methylation, Cellular heterogeneity, Nonnegative matrix factorization, Single-cell methylome

## Abstract

**Background:**

Numerous cell types can be identified within plant tissues and animal organs, and the epigenetic modifications underlying such enormous cellular heterogeneity are just beginning to be understood. It remains a challenge to infer cellular composition using DNA methylomes generated for mixed cell populations. Here, we propose a semi-reference-free procedure to perform virtual methylome dissection using the nonnegative matrix factorization (NMF) algorithm.

**Results:**

In the pipeline that we implemented to predict cell-subtype percentages, putative cell-type-specific methylated (pCSM) loci were first determined according to their DNA methylation patterns in bulk methylomes and clustered into groups based on their correlations in methylation profiles. A representative set of pCSM loci was then chosen to decompose target methylomes into multiple latent DNA methylation components (LMCs). To test the performance of this pipeline, we made use of single-cell brain methylomes to create synthetic methylomes of known cell composition. Compared with highly variable CpG sites, pCSM loci achieved a higher prediction accuracy in the virtual methylome dissection of synthetic methylomes. In addition, pCSM loci were shown to be good predictors of the cell type of the sorted brain cells. The software package developed in this study is available in the GitHub repository (https://github.com/Gavin-Yinld).

**Conclusions:**

We anticipate that the pipeline implemented in this study will be an innovative and valuable tool for the decoding of cellular heterogeneity.

## Introduction

DNA methylation plays a key role in tissue development and cell specification. As the gold standard for methylation detection, bisulfite sequencing has been widely used to generate genome-wide methylation data and computational efforts have been made to meet the statistical challenges in mapping bisulfite-converted reads and determining differentially methylated sites [1–4]. Methylation data analysis has been extended from simple comparisons of methylation levels to more sophisticated interpretations of methylation patterns embedded in sequencing reads, which are referred to as the combinatory methylation statuses of multiple neighboring CpG sites [5].

Through multiple bisulfite sequencing reads mapped to a given genome locus, methylation entropy can be calculated as a measurement of the randomness, specifically the variations, of DNA methylation patterns in a cell population [6]. It was soon realized that such variations in methylation patterns could have resulted from methylation differences: (1) among different types of cells in a mixed cell population, (2) between the maternal and paternal alleles within a cell, or (3) between the CpG sites on the top and bottom DNA strands within a DNA molecule [7–9]. The genome-wide hairpin bisulfite sequencing technique was developed to determine strand-specific DNA methylation, i.e., methylation patterns resulting from (3). The methylation difference between two DNA strands is high in embryonic stem cell (ESC) but low in differentiated cells [8]. For instance, in human brain, the chances of four neighboring CpG sites having an asymmetric DNA methylation pattern in a double-stranded DNA molecule are less than 0.02% [10]. Allelic DNA methylation, i.e., methylation patterns resulting from (2), was found to be limited in a small set of CpG sites. In the mouse genome, approximately two thousand CpG sites were found to be associated with allele-specific DNA methylation [11]. Thus, cellular heterogeneity could be a primary source of the variations in DNA methylation patterns. This often leads to bipolar methylation patterns, meaning that genome loci are covered both with completely methylated reads and completely unmethylated reads simultaneously in bulk methylomes. Such bipolar methylated loci can be detected using nonparametric Bayesian clustering followed by hypothesis testing and were found to be highly consistent with the differentially methylated regions identified among purified cell subsets [12]. For this reason, these loci are called the putative cell-type-specific methylated (pCSM) loci. They were further demonstrated to exhibit methylation variation across single-cell methylomes [13].

An appropriate interpretation of methylome data derived from bulk tissues requires consideration of methylation variations contributed by diverse cellular compositions. With the existing reference methylomes for different types of cells, it is possible to estimate cell ratios in a heterogeneous population with known information about the cell types. For instance, cell mixture distributions within peripheral blood can be assessed using constrained projection, which adopts least-squares multivariate regression to estimate regression coefficients as the ratios for cell types [14]. More recent studies suggest that non-constrained reference-based methods are robust across a range of different tissue types [15] and Bayesian semi-supervised methods may construct cell-type components in a way that each component corresponds to a single-cell type [16]. For reference-based algorithms, prior knowledge of cell composition and cell-specific methylation markers is critical [17]. To overcome these issues, principal component analysis (PCA) was adopted by ReFACTor for the correction of cell-type heterogeneity [18], and nonnegative matrix factorization (NMF) was adopted by MeDeCom to recover cell-type-specific latent methylation components [19]. However, the performance of such reference-free cell-type deconvolution tools relies heavily on model assumptions [20]. Recently, the development of single-cell DNA methylation sequencing techniques generated a growing number of methylomes at unprecedented resolution, providing new opportunities to explore cellular diversity within cell populations [21–27]; yet, no attempt has been taken to make use of single-cell methylomes for cell-type deconvolution analysis.

In this study, we propose a semi-reference-free, NMF-based pipeline to dissect cell-type compositions for methylomes generated from bulk tissues. This pipeline takes advantage of pCSM segments that exhibit bipolar methylation patterns in methylomes generated from bulk tissues or among single-cell methylomes. To overcome the shallow depth of whole-genome bisulfite sequencing, weighted gene co-expression network analysis (WGCNA) was modified to cluster pCSM loci. PCA was performed to select eigen-pCSM loci, which are representative loci for clusters of pCSM loci. To evaluate the performance of eigen-pCSM loci selected in cell-type deconvolution, over 3000 brain single-cell methylomes were mixed in random proportions in simulation studies to create synthetic methylomes. The pipeline implemented in this study provides an accurate estimation of cell-type composition on both synthetic methylomes and bulk methylomes from five neuronal cell populations.

## Results

### Virtual methylome dissection based on eigen-pCSM loci

**Fig. 1:**
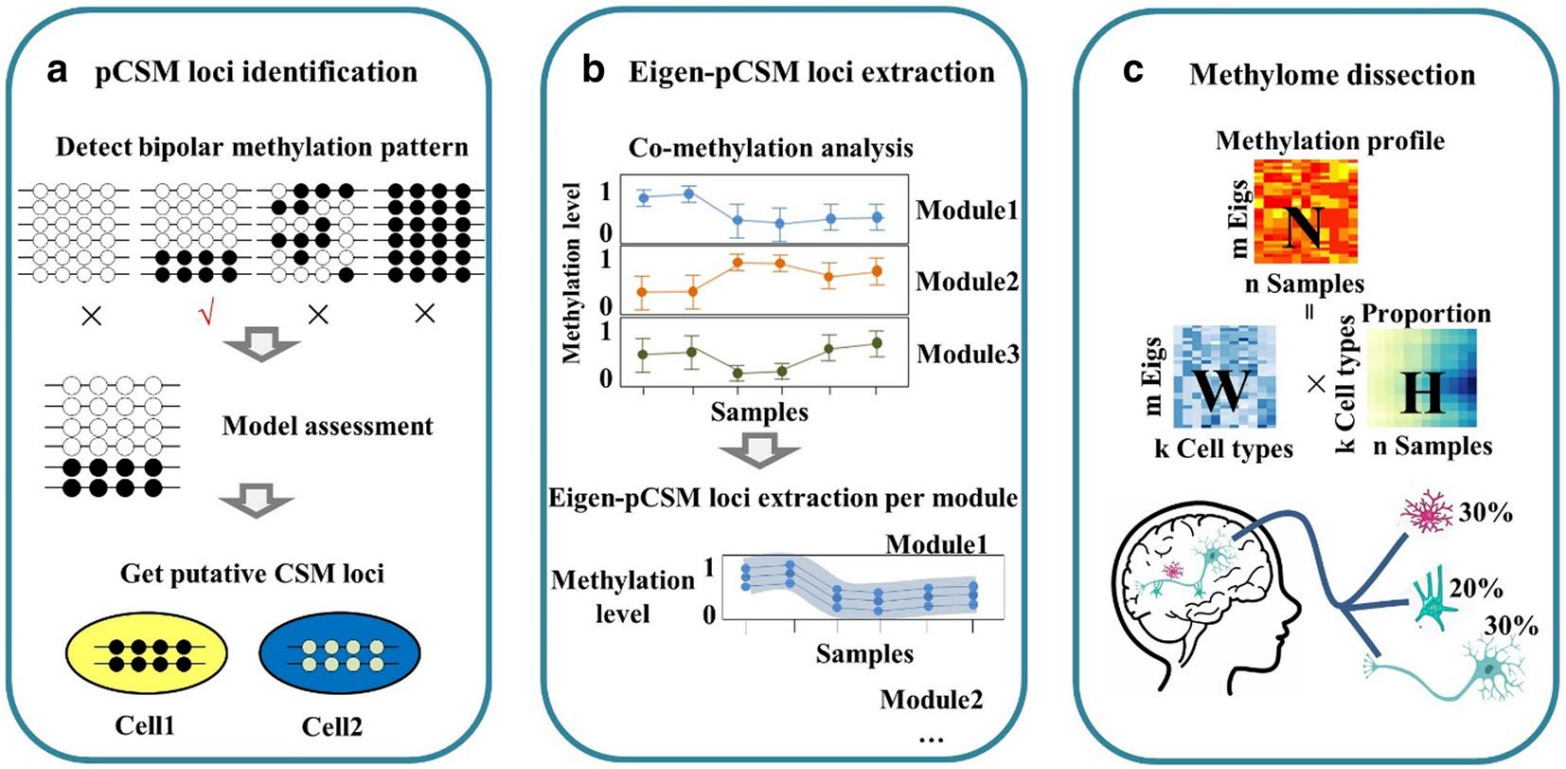
A three-step process to perform methylome dissection using eigen-pCSM loci. **a** In the first step, bipolar 4-CG segments are identified and a nonparametric Bayesian clustering algorithm is used for the determination of pCSM loci. **b** In the second step, co-methylation analysis is performed by *k*-means clustering coupled with WGCNA analysis. In each co-methylation module, PCA analysis is performed to pick the eigen-pCSM loci as a representative for the whole module. **c** In the third step, methylome dissection is performed by nonnegative matrix factorization (NMF), where matrix *N* stands for the raw methylation profile and is decomposed into two matrices, *W* and *H*. Matrix *W* represents the methylation profile of cell components, and matrix *H* represents the proportion of cell components

To perform virtual methylome dissection, we introduced a three-step pipeline (Fig. 1). In the first step, pCSM loci were determined for target methylomes, which were generated from various sources including tissues, sorted cells, or single cells. The key issue in this step was to efficiently distinguish cell-type-specific DNA methylation events from stochastic methylation events. Using the hairpin bisulfite sequencing approach, we observed that 5% of CpG sites were asymmetrically methylated, but the frequencies of asymmetric methylation events decreased more than 200 times from approximately 5% for a single CpG to 0.02% for a sliding window of a 4-CpG genomic segment [10]. Therefore, in our proposed pipeline, the methylation patterns of 4-CpG genomic segments were determined from each bisulfite-converted sequencing read to minimize the influence of asymmetric DNA methylation. For all 4-CpG segments mapped to a given genomic loci, the variation in their methylation patterns was subjected to nonparametric Bayesian clustering followed by hypothesis testing to infer bipolar methylated loci [12]. After the filtering of allelic-specific methylated regions and merging overlapping segments, pCSM loci were collected for co-methylation analysis. In the second step, eigen-pCSM loci, representing pCSM clusters with distinct methylation profiles, were determined by WGCNA clustering and PCA analysis. In the third step, target methylomes were decomposed with eigen-pCSM loci using the NMF algorithm. The methylation matrix of eigen-pCSM loci in all samples was decomposed into a product with two matrices: one for the methylation profiles of estimated cell types and the other for the cell-type proportions across all samples.

Mammalian brain consists of many functionally distinct cell subsets that can contribute to diverse DNA methylation patterns on loci with cell subset-specific methylation. In particular, diverse subpopulations of neurons and glial cells can often be found even within a given brain region [28]. To demonstrate the effectiveness of our procedure, we performed two distinct analyses using synthetic methylomes derived from brain single cells and methylomes from brain-sorted cells.

### pCSM loci predicted with brain single-cell methylomes

Our first case study took advantage of recent brain single-cell methylomes generated for 3377 neurons derived from mouse frontal cortex tissue [21] (Additional file 1: Table S1). Following our previous procedure for single-cell methylome analysis [13], we determined the pCSM loci from each single-cell methylome. Briefly, for each methylome, we scanned the sequence reads one by one to identify genomic segments with methylation data for four neighboring CpG sites. To facilitate pCSM identification from the 4,326,935 4-CG segments identified, we first selected 1,070,952 pCSM candidates that were completely methylated in at least one neuron but also completely unmethylated in another. We next applied the beta mixture model to the methylation patterns in single neurons for these candidates segments [13]. 921,565 segments were determined to be pCSM segments with bipolar distributed methylation profiles, while the rest (149,387 segments) had heterogeneous methylation patterns among neurons.

**Fig. 2:**
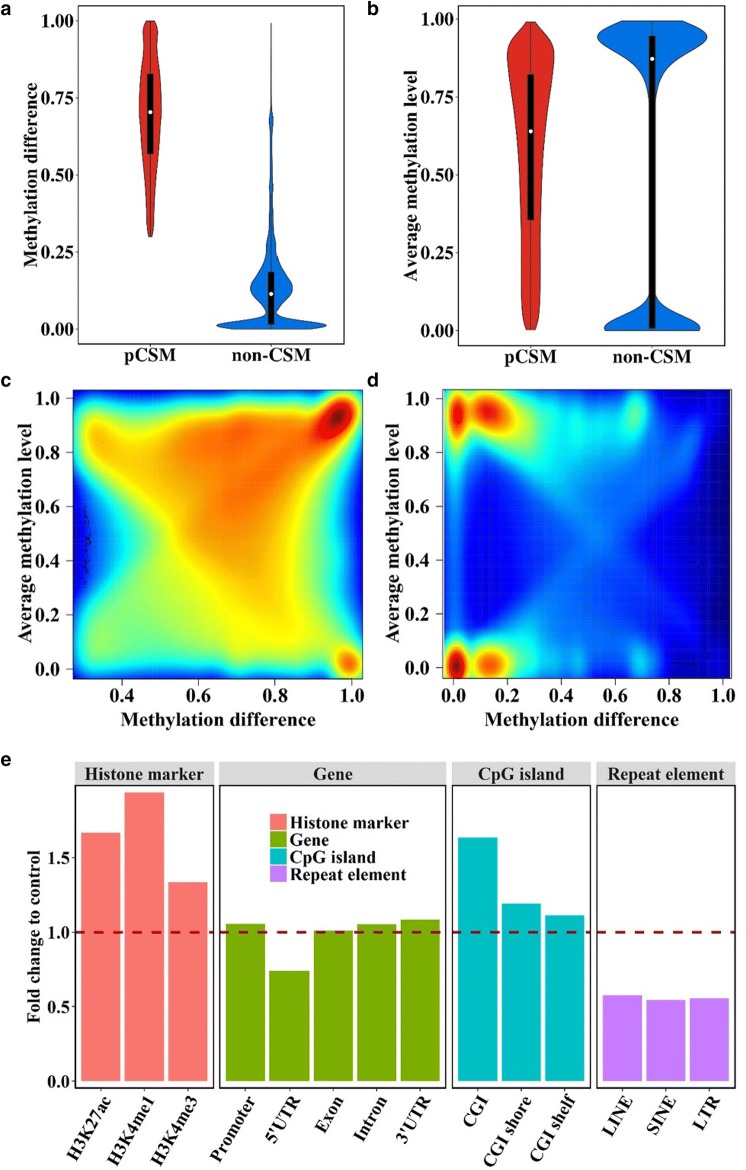
pCSM segments reflected methylation heterogeneity. **a** Distribution of methylation differences between cell subsets classified with pCSM and non-CSM segments. **b** Average methylation levels of pCSM segments and non-CSM segments across single cells. **c**, **d** Relationship between methylation level and methylation difference of pCSM segments (**c**) and non-CSM segments (**d**). The color indicates the densities of pCSM segments or non-CSM segments from low (blue) to high (red). **e** The distribution of pCSM loci across various genomic features compared to those of control regions

To gain a better understanding of pCSM, we analyzed several features of these 921,565 pCSM segments using the leftover 3,405,370 non-CSM segments from the starting 4,326,935 segments as controls. According to the methylation status of each 4-CG segment, we assigned the neurons into two subsets, hypermethylated and hypomethylated, and calculated the methylation difference of each 4-CG segment between the two cell subsets. For non-CSM segments with all methylated reads or unmethylated reads, only one cell subset could be identified, and thus, the methylation difference was set as zero. As expected, pCSM segments showed large methylation differences between the two cell subsets with an average of 0.70, while the average methylation difference for non-CSM segments was only 0.11 (Fig. 2a). The average methylation levels of pCSM segments among cells were broadly distributed, while the non-CSM segments tended to be either hypermethylated or hypomethylated (Fig. 2b). Some pCSM segments had average methylation levels approaching 1 or 0, but their bipolar methylation patterns allowed the splitting of cells into two groups with a methylation difference close to 1 (Fig. 2c). In contrast, the majority of either hypermethylated or hypomethylated non-CSM segment cells split into two groups with a methylation difference less than 0.2 (Fig. 2d).

To further explore the functional characteristics of pCSM segments, we merged the overlapped pCSM segments into 347,889 loci (Additional file 2: Table S2) and integrated them with brain histone modification maps. We observed that these pCSM loci were enriched at H3K27ac, H3K4me, and H3K4me3 peaks and CpG islands with 1.63-, 1.93-, 1.28-, and 1.52-fold increases, respectively (Fig. 2e). In addition, pCSM loci were depleted from repeat regions including SINE, LINE, and LTR. This result suggested that pCSM loci might play important regulatory roles in the brain. For the pCSM loci that overlapped with histone marks for enhancers or promoters, we identified their adjacent genes for functional enrichment analysis using the GREAT analysis tools [29]. As shown in Additional file 3: Figure S1, genes associated with these pCSM loci are significantly enriched in the functional categories for brain development, such as “regulation of synaptic plasticity” and “metencephalon development.” Altogether, these results indicate that pCSM loci showing bipolar methylation among neurons may play important roles in the epigenetic regulation of brain development.

### Synthetic methylome: eigen-pCSM loci determination and virtual methylome dissection by NMF

**Fig. 3:**
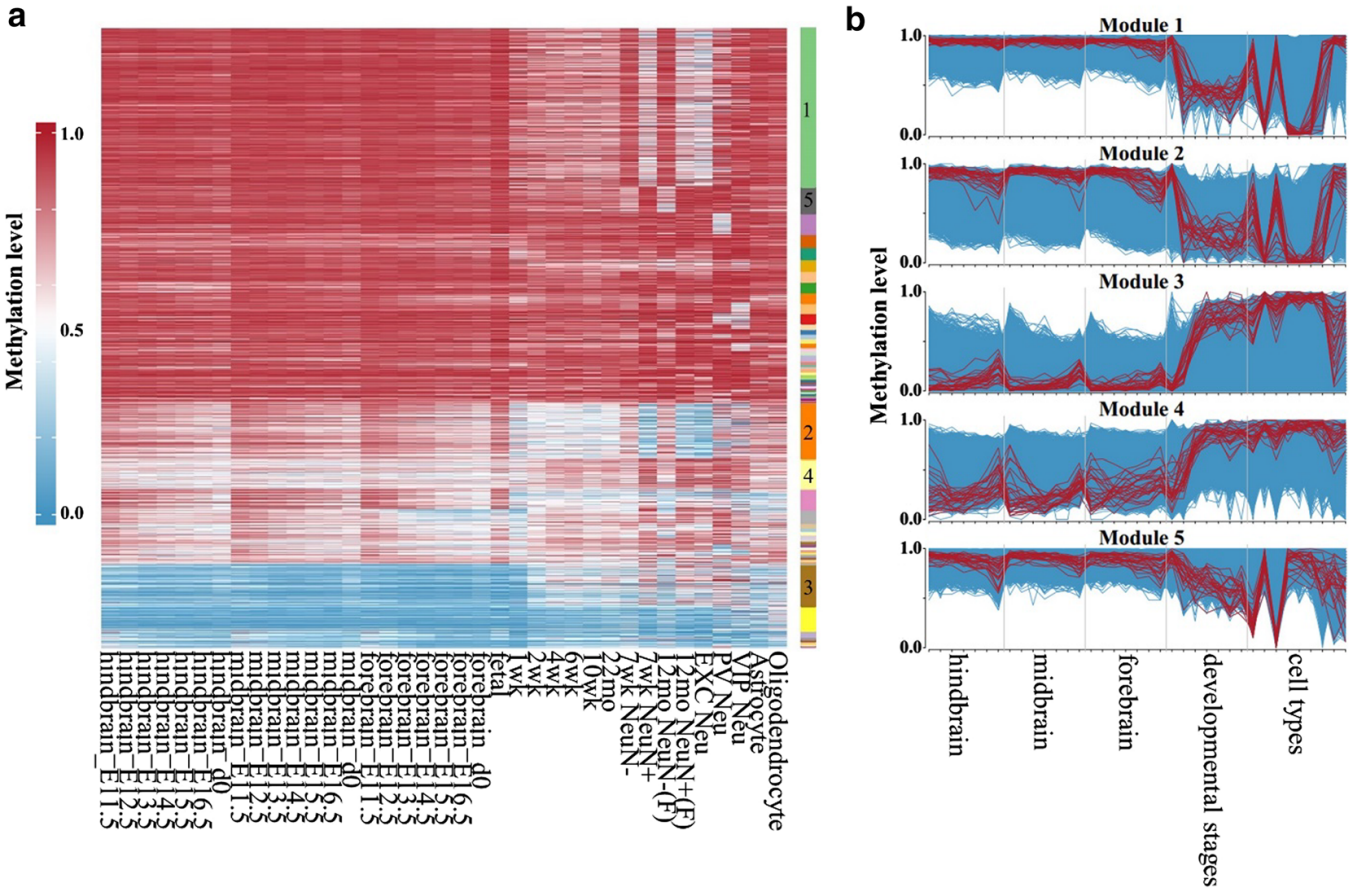
Co-methylation analysis to extract eigen-pCSM loci. **a** Heatmap of the methylation level of pCSM loci across brain methylomes. The methylation levels were represented by color gradient from blue (unmethylation) to red (full methylation). The color key in the right panel represents co-methylation modules. **b** Methylation profiles of the top five co-methylation modules. Each blue line represents the methylation level of pCSM loci across brain methylomes, the red lines represent the methylation level of eigen-pCSM loci picked by PCA analysis in each module, and 10% eigen-pCSM loci with the maximal loadings in PC1 were shown

In the previous study [21], a total of 3377 neurons were clustered into 16 neuronal cell types including mL2.3, mL4, mL5.1, mL5.2, mL6.1, mL6.2, mDL.1, mDL.2, mDL.3, and mIn.1 for excitatory neurons and mVip, mPv, mSst.1, mSst.2, mNdnf.1, and mNdnf.2 for inhibitory neurons. Such single-cell methylomes with assigned cell-type information provide ideal training and test sets to examine our approach. By merging single-cell methylomes within each cluster, we first created 16 artificial methylomes as references for distinct cell types. These 16 reference methylomes were then mixed in random proportions to create synthetic methylomes. To overcome the low read depth at each genomic locus, we performed clustering analysis to extract eigen-pCSM loci from the synthetic methylomes (Fig. 1b). To identify co-methylated modules, we collected a total of 61 mouse methylomes across all brain development stages and cell types (Additional file 1: Table S1). Based on the methylation profiles of pCSM loci in these brain methylomes, co-methylation analysis was performed through *k*-means clustering followed by weighted correlation network analysis [30] (Fig. 3a). For each co-methylation module, PCA analysis was performed to select a subset of pCSM loci as the eigen-pCSM loci representing the methylation trend (Fig. 3b).

**Fig. 4:**
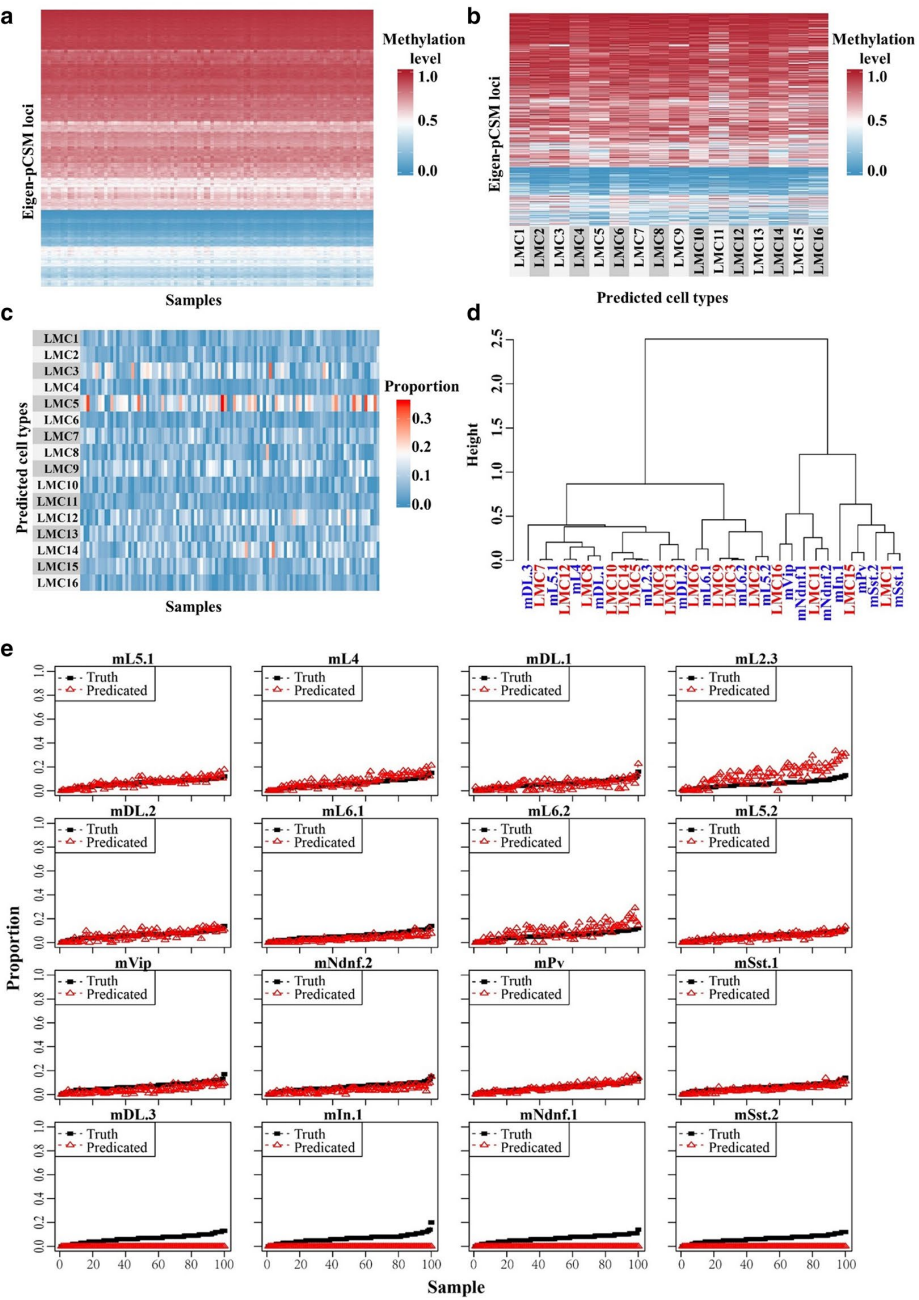
Virtual methylome dissection based on eigen-pCSM loci. **a** Methylation profiles of eigen-pCSM loci, with each row representing an eigen-pCSM locus and each column representing one synthetic methylome. **b** Methylation profiles of NMF predicted cell types, with each row representing an eigen-pCSM loci and each column representing an NMF predicted cell type. **c** Heatmap of cell proportions predicted with NMF across all samples, with each row representing an NMF predicted cell type and each column representing a sample. The proportions were represented by color gradient from blue (low) to red (high). **d** Clustering analysis of cell types predicted by NMF and 16 reference methylomes. **e** Recovery of the mixing ratios for 16 neuronal cell types. The reference cell types that could not be unambiguously assigned to an LMC were considered as failures in prediction with a ratio of zero. In each line plot, the synthetic samples are sorted by ascending true mixing proportion

We simulated 100 synthetic methylomes composed of 16 reference methylomes in various ratios. The number of LMCs (*k* = 16) was determined according to prior knowledge, and the regularizer shifts’ parameter (*λ* = 1e−04) was selected via cross-validation provided in the MeDeCom package (Additional file 3: Figure S2A). Each synthetic methylome was dissected into multiple latent DNA methylation components representing the hypothetic origins of the 16 reference methylomes (Fig. 4a, b) with their proportions determined (Fig. 4c). We further assigned the cell types predicted by NMF to the aforementioned 16 reference methylomes via clustering analysis (Fig. 4d). Corresponding to the decomposed cell types, the proportions of cell types predicted with NMF were also accurately reproduced (Fig. 4e) with a mean absolute error (MAE) of 0.037, which serves as a measure for the precision of the proportions of LMCs predicted by NMF. A high level of Pearson’s correlations with a range from 0.82 to 1.00 was observed between the 12 immediately grouped reference neuronal types (i.e., mL5.1, mL4, mDL.1, mL2.3, mDL.2, mL6.1, mL6.2, mL5.2, mVip, mNdnf.2, mPv, and mSst.1) and the predicted cell types (Additional file 3: Figure S2B). The other four types of neuronal cells, including mDL.3, mIn.1, mNdnf.1, and mSst.2, were not decomposed from synthetic methylomes. The percentages of these four types of neurons only account for a small fraction (< 1.7%) of the 3377 neurons sequenced (Additional file 3: Figure S2C). The mapped reads for these four types were very limited (Additional file 3: Figure S2D). Thus, the methylation features of these four types may not be fully represented by the small number of pCSM loci identified (Additional file 3: Figure S2E). Since the proportions of the 16 cell types followed a uniform distribution in the simulation study (Additional file 3: Figure S2F), the failure in cell component decomposition is likely due to insufficient information in the eigen-pCSM loci to distinguish these four types of neurons from the others. This indicates that our procedure could have a detection limit for the rare cells. Another possibility is that some of the components had the unidentified cell types as their second-best matches. Therefore, missing just a few population-specific loci, e.g., due to poor coverage, could be the reason behind this loss of identifiability.

**Fig. 5:**
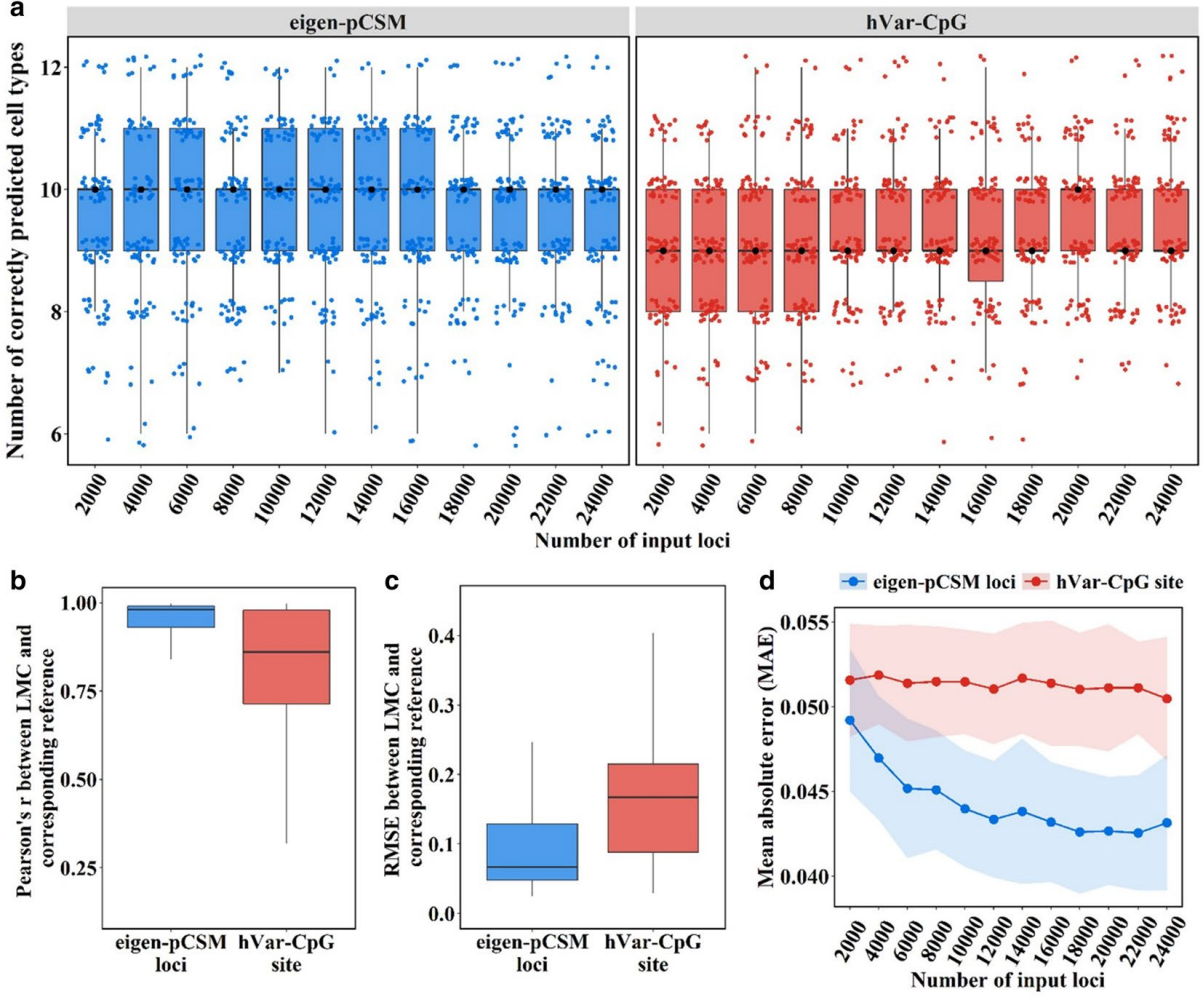
Performance of virtual methylome dissection based on eigen-pCSM loci and hVar-CpG sites. **a** Number of correctly predicted cell types in each simulation. **b** Pearson correlation coefficient between LMCs and their corresponding reference methylome. **c** The root-mean-square error (RMSE) between LMCs and their corresponding reference methylome. **d** Mean absolute error (MAE) between NMF predicted proportions and real proportions, with the dot showing the mean MAE and the shade showing the standard deviation of the MAE in 100 simulations

In a previous study [19], highly variable CpG (hVar-CpG) sites, i.e., CpG sites with high sample-to-sample methylation variance, were proposed for the dissection of bulk methylomes. We next performed simulations 100 times with 2000 to 24,000 hVar-CpG sites or with pCSM loci to compare the classification accuracy using hVar-CpG sites *vs* pCSM loci. For the 16 cell types, the eigen-pCSM-loci-based method accurately assigned ten on average, while the hVar-CpG-sites-based method only predicted nine on average (Fig. 5a). Compared to the hVar-CpG-sites-based method, the eigen-pCSM-loci-based method exhibited a higher correlation and lower root-mean-square error (RMSE) between LMCs and their corresponding reference methylomes (Fig. 5b, c). In addition, a lower MAE was achieved with the increasing number of eigen-pCSM loci from each module. However, such an improvement could not be achieved by using additional hVar-CpG sites (Fig. 5d).

### Brain methylome: virtual methylome dissection for neuronal cells

To examine whether the proposed virtual methylome dissection approach can be applied to the methylomes generated from tissue samples, we re-analyzed five brain methylomes derived from sorted nuclei including excitatory (EXC) neurons, parvalbumin (PV) expressing fast-spiking interneurons, vasoactive intestinal peptide (VIP) expressing interneurons [31], and mixed neurons from the cortex’s of 7-week (7wk NeuN+) and 12-month (12mo NeuN+) mice [32]. These five methylomes were analyzed separately and together as a mixed pool (Additional file 3: Figure S3A). 19,091 to 212,218 pCSM segments were identified in the six methylomes, accordingly. Among the 212,218 pCSM segments identified in the mixed pool, 118,409 segments showed differential DNA methylation states across the five neuronal samples; the other 93,809 pCSM segments were found to be pCSM segments within the five methylomes (Additional file 3: Figure S3B). Since a significant number of pCSM segments can be identified from pooled samples to capture differences among sorted cells (Additional file 3: Figure S3B), it is a better strategy to pool methylomes from sorted cells for pCSM loci identification, particularly when methylomes have a low read depth.

Next, we asked whether the pCSM segments identified from the pooled methylome could reflect the cell-type-specific methylation pattern derived from single-cell methylomes. Interestingly, we found that the pCSM segments identified from the pooled methylome were significantly overlapped with those identified using single-cell methylomes (Additional file 3: Figure S3C). This indicates that the cell-type-specific methylated loci determined with single-cell methylomes could also be detected using a bulk methylome. In addition, pCSM loci identified from the pooled methylome (Additional file 4: Table S3) were enriched at enhancer histone markers and CpG islands, but were depleted from promoter, 5′UTR, and repeat elements (Additional file 3: Figure S3D).

**Fig. 6:**
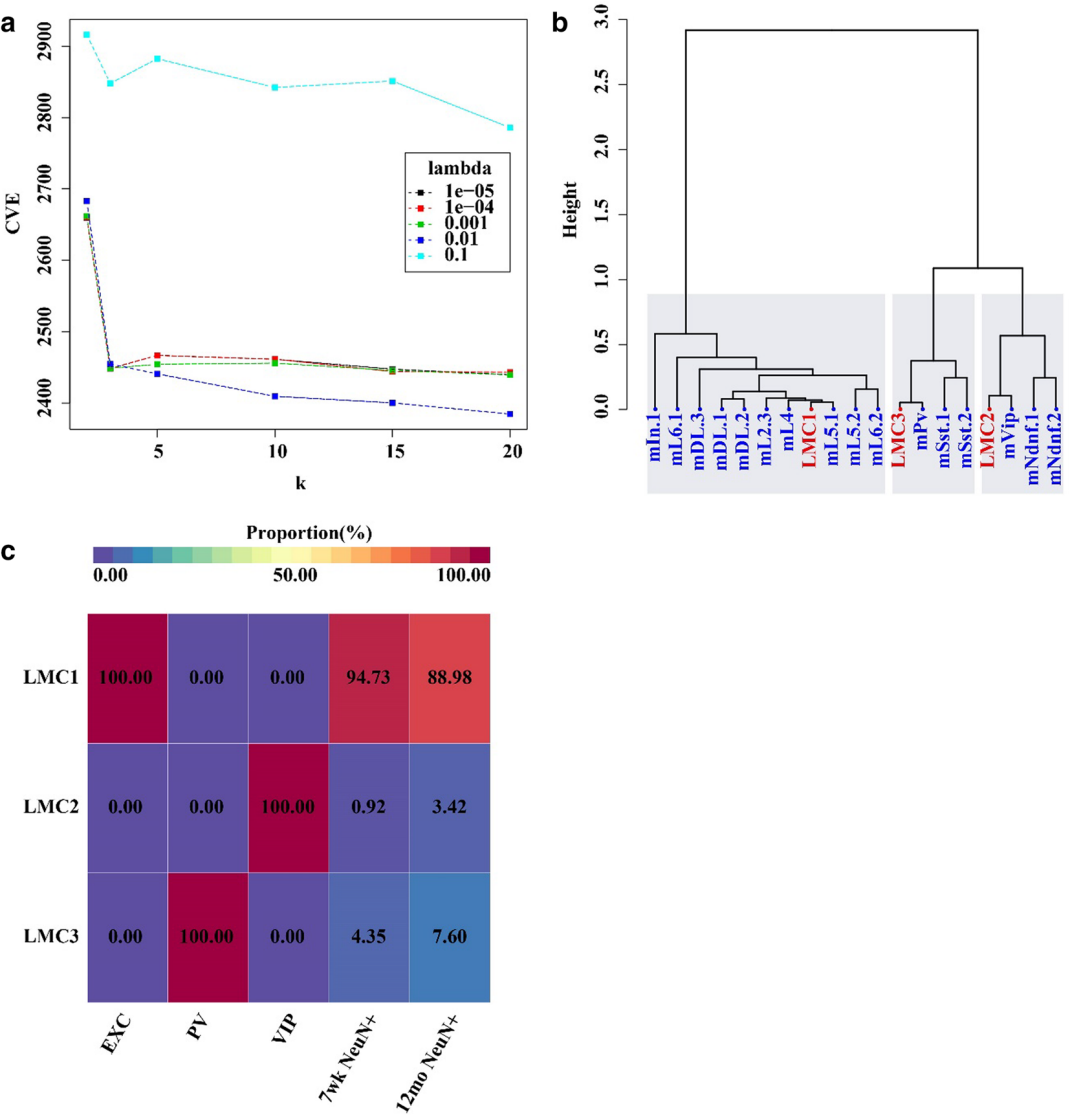
Methylome virtual dissection of five neuronal sorted cell populations. **a** Selection of parameters *k* and *λ* by cross-validation provided by MeDeCom Package. **b** Clustering analysis of predicted cell types and reference cell types when *k* = 3, with the red nodes representing the predicted cell types and the blue nodes representing the reference cell types from single-cell methylomes. **c** Predicted proportions of each LMC in five datasets

To further explore the composition of the five neuronal cell populations, we performed methylome virtual dissection based on pCSM loci identified from the pooled methylome. Following the aforementioned procedure, we performed co-methylation analysis and extracted eigen-pCSM loci from each module. An NMF model was performed with 20,000 eigen-pCSM loci selected to decompose the five methylomes. The cross-validation error showed a substantial change at *k *≥ 3 (Fig. 6a), which indicated the existence of at least three major epigenetically distinct cell components, i.e., LMCs. We then examined the factorization results and compared the three main LMCs at *k *= 3 and *λ *= 10^−5^ to the single-cell reference profiles. Clustering analysis showed that the reference profiles of EXC, PV, and VIP neurons are related to LMC1, LMC3, and LMC2, respectively (Fig. 6b). In addition, we found that the samples of EXC, PV, and VIP neurons have high purity (Fig. 6c). Although the cellular composition of NeuN+ cells is unknown and depends highly on the cell sorting procedure, about 70–85% of mouse cortical neurons are excitatory with 6–12% PV neurons and 1.8–3.6% VIP neurons [31, 33]. In our study, the 7-week NeuN+ sample was predicted to have a mixture of 94.73% excitatory neurons, 4.35% PV neurons, and 0.92% VIP neurons. The 12-month NeuN+ sample was predicted to consist of 88.98% excitatory neurons, 7.6% PV neurons, and 3.42% VIP neurons. Considering the fact that inhibitory neurons have been reported as more likely to be depleted during the NeuN sorting procedure [34], our predictions were largely consistent with the known composition of mouse cortical neurons. Altogether, these results indicate that pCSM loci may serve as excellent predictors to decompose bulk methylomes.

## Discussion

In this study, we implemented an analysis pipeline to predict the composition of cell subtypes in bulk methylomes. To our knowledge, this is the first endeavor to systematically analyze the variation in DNA methylation patterns to infer pCSM loci as inputs for the NMF model. Application of synthetic methylomes that are simulated based on single-cell methylomes and methylomes derived from sorted cells demonstrated that our approach is efficient and has high prediction accuracy. Our procedure is semi-reference free. The clustering of pCSM loci to identify representative eigen-pCSM loci depends on the methylomes collected. With rapidly accumulating methylome data, such a method will gain power and can be widely used to explore cell heterogeneity during tissue development and disease progression.

## Materials and methods

### Analyses of single-nucleus methylcytosine sequencing (snmC-seq) datasets

Single-nucleus methylcytosine sequencing datasets of 3377 neurons from 8-week-old mouse cortex (GSE97179) were downloaded from the Gene Expression Omnibus (GEO). These datasets were analyzed following the processing steps provided in a previous study [21]: (1) Sequencing adaptors were first removed using Cutadapt v2.1 [35], (2) trimmed reads were mapped to the mouse genome (GRCm38/mm10) in single-end mode using Bismark v0.16.3 [1], with the pbat option activated for mapping R1 reads [21], (3) duplicated reads were filtered using picard-tools v2.0.1, (4) non-clonal reads were further filtered by minimal mapping quality (MAPQ ≥ 30) using samtools view [36] with option −q30, and (5) methylation calling was performed by Bismark v0.16.3.

### Identification of pCSM loci from snmC-seq datasets

pCSM loci were determined from single-cell methylomes with a similar procedure to what was provided in a previous study [13]. Briefly, for each snmC-seq dataset, all segments with four neighboring CpG sites in any sequence read were extracted from autosomes, and the corresponding methylation patterns were recorded. The 4-CpG segments that overlapped with known imprinted regions [11] were excluded in subsequent steps. To ensure statistical power for the identification of pCSM loci, segments covered by at least ten single-cell methylomes were retained for further analysis. The remaining 4-CG segments covered by at least one completely methylated cell and one completely unmethylated cell in such genomic loci were identified as CSM loci candidates. From these candidates, a beta mixture model [13] was used to infer pCSM loci, by which cells that covered the same segment could be grouped into hypomethylated and hypermethylated cell subsets. The segments with methylation differences between hypomethylated and hypermethylated cell subsets over 30% and adjusted p values less than 0.05 were then identified as the pCSM loci.

### Analyses of whole-genome bisulfite sequencing datasets

Sequencing adaptors and bases with low sequencing quality were first trimmed off using Trim Galore v0.4.4. The retained reads were then mapped to the mouse reference genome (GRCm38/mm10) using Bismark v0.16.3. Duplicated reads were removed using deduplicate_bismark. Lastly, methylation calling was performed by Bismark v0.16.3.

### Identification of pCSM loci from WGBS datasets

pCSM loci were identified from WGBS datasets following a strategy described previously [10] with slight modifications. Genomic segments with four neighboring CpGs were determined within each sequence read. Such 4-CpG segments covered with at least ten reads were retained for further identification of bipolar methylated segments. A nonparametric Bayesian clustering algorithm [12] was performed to detect bipolar methylated segments that were covered by at least one completely methylated and one completely unmethylated read concurrently. Bipolar segments in chromosome X, Y, and known imprinted regions [11] were excluded from further analysis.

### Genome annotation and gene ontology analysis

Genomic features were downloaded from the UCSC Genome database [37], including annotation for gene structure, CpG islands (CGI), and repeat elements in mm10. Promoters were defined as 2 kb regions upstream of transcription starting sites (TSS). CGI shores were defined as 2 kb outside of the CGI, and CGI shelves were defined as 2 kb outside of the CGI shores. The broad peaks of histone modifications H3K4me1, H3k4me3, and H3K27ac for 8-week mouse cortex were obtained from the ENCODE Project [38] (with accession GSM769022, GSM769026, and GSM1000100, respectively) and lifted from mm9 to mm10 using UCSC LiftOver tools. GO enrichment analysis for pCSM loci enriched in histone peaks was performed by the GREAT tool V3.0.0 [29] using default settings.

### Co-methylation, eigen-pCSM loci extraction, and NMF analyses for virtual methylome dissection

A two-step clustering approach was adopted for co-methylation analysis. First, *k*-means clustering analysis was performed to divide pCSM loci into hypo/mid/hypermethylation groups. For each *k*-means cluster, the R package WGCNA v1.61 [30] was used to identify co-methylation modules of highly correlated pCSM loci. Briefly, for a given DNA methylation profile, a topological overlap measure (TOM) was used to cluster pCSM loci into network modules. The soft-thresholding power was determined with the scale-free topology. Network construction and module determination were performed using the “blockwiseModules” function in WGCNA, and the network type was set to “signed” during network construction to filter the negatively correlated pCSM loci within one module. PCA analysis was performed to select a subset of pCSM loci with the maximal loadings in PC1 as eigen-pCSM loci for the corresponding module.

The R package MeDeCom V0.2 [19] was used to dissect the methylomes using NMF analysis. A matrix with eigen-pCSM loci in rows and samples in columns can be decomposed into the product of two matrices: one representing the profile of predicted cell types with eigen-pCSM loci in rows and cell types in columns and the other containing the proportion of predicted cell types in each sample with cell types in rows and samples in columns. Two parameters need to be artificially set in NMF analysis, i.e., the number of cell types *k*, and the regularizer shifts’ parameter *λ*, by which the estimated matrix of methylation patterns toward biologically plausible binary values close to zero (unmethylated) or one (methylated). *k* is dictated by prior knowledge on the input methylomes. In the case that no prior knowledge of cell composition is available for the input methylomes, both *k* and *λ* may be selected via cross-validation as suggested in the MeDeCom package.

### Cell mixture methylome synthesis and virtual methylome dissection simulation

First, 16 artificial methylomes were created as references by merging single-cell methylomes of each neuronal cell type identified in a previous study [21]. Then, the simulated methylomes were generated by mixing the reference methylomes with random proportions. In each simulation, 100 methylomes were synthesized, based on which virtual methylome dissection was performed using the profiles of the eigen-pCSM loci in these 100 methylomes. To identify cell components from the dissection results, clustering analysis was performed on the dissected LMCs and 16 reference neuronal cell types, and the LMCs unambiguously matched to one of the reference neuronal cell types were considered to be recognized. The RMSE between LMCs and their matched reference methylomes was calculated to evaluate the recovery of reference methylomes by the following formula:

$$ {\text{RMSE}} = \sqrt {\frac{{\mathop \sum \nolimits_{i = 1}^{N} (m_{i} - \widehat{{m_{i} }})^{2} }}{N}} $$

where each pair of $m$ and $\widehat{m}$ denotes the true methylation level (*m*) of one genomic loci in the reference methylation and the estimated methylation level ($\widehat{m}$) of that loci in the corresponding predicted cell component. *N* denotes the number of loci.

To evaluate the recovery of the mixing proportions, the MAE between true proportions of neuronal cell types and the estimated proportions of recognized cell components was calculated by the following formula:

$$ {\text{MAE}} = \frac{{\mathop \sum \nolimits_{i = 1}^{16} \left| {p_{i} - \widehat{{p_{i} }}} \right|}}{16} $$

where each pair of *p* and $\widehat{p}$ denotes the true proportion (*p*) of one reference neuronal cell type and the estimated proportion ($\widehat{p}$) of its corresponding predicted cell component. The proportions of the estimated cell components that cannot be mapped to the true cell types were set to zero. For comparison, a parallel analysis was also performed using 2000 to 24,000 hVar-CpG sites with the maximal sample-to-sample variation.

## Supplementary information

**Additional file 1: Table S1.** A summary of data source for datasets derived from mouse brain and sorted neurons.

**Additional file 2: Table S2.** Genomic coordinate (mm10 based) of pCSM loci identified from single-cell brain methylomes.

**Additional file 3: Figure S1.** Functional enrichment of genes with pCSM loci overlapped with enhancer or promoter histone marks. **Figure S2.** Virtual methylome dissection using eigen-pCSM loci. **A**) Selection of parameter *λ* by cross-validation. **B**) Pearson’s correlation coefficient between real cell types and NMF predicted cell types. **C**) The number of cells in each neuronal cell types identified by Luo et al. The percentage of each neuronal type in 3377 neurons sequenced is shown at the top of each bar. **D**) The number of mapped reads in each neuronal cell type. The fraction of reads mapped in each neuronal type accounts for all mapped reads in 3377 neurons is shown at the top of each bar. **E**) The fraction of the pCSM loci covering each cell type. **F**) The synthetic proportions of each neuronal cell type. The error bar shows the standard deviation of the synthetic proportions in 100 methylomes. **Figure S3.** Characteristics of pCSM loci identified from brain methylomes**. A)** A sketch map of pooling samples. **B)** Number of pCSM segments identified from neuronal and pooled methylome. “Vanished” represents the segments identified as pCSM segments within each neuronal cell population but identified as non-CSM segments in pooled sample. “Emerged” represents the segments identified as pCSM segments in pooled sample but identified as non-CSM segments within each individual cell population. “Derived” represents the segments identified as pCSM segments in both pooled sample and at least one neuronal cell population. **C)** Venn plot shows the overlap between pCSM segments identified from single-cell methylomes and those identified from the pooled methylome. **D)** The distribution of pCSM loci across various genomic features compared to those of control regions.

**Additional file 4: Table S3**. Genomic coordinate (mm10 based) of pCSM loci identified from bulk brain methylomes.

## Data Availability

Source code for pCSM loci identification and eigen-pCSM loci extraction is available at https://github.com/Gavin-Yinld/csmFinder and https://github.com/Gavin-Yinld/coMethy, respectively.
