# Supplementary material for: Virtual methylome dissection facilitated by single-cell analyses
Source: Epigenetics Chromatin. 2019 Nov 11;12:66. doi: 10.1186/s13072-019-0310-9 (PMC6844058; doi:10.1186/s13072-019-0310-9)
Supplement: Supplementary file 3 — Additional file 3: Figure S1. Functional enrichment of genes with pCSM loci overlapped with enhancer or promoter histone marks. Figure S2. Virtual methylome dissection using eigen-pCSM loci. A) Selection of parameter λ by cross-validation. B) Pearson’s correlation coefficient between real cell types and NMF predicted cell types. C) The number of cells in each neuronal cell types identified by Luo et al. The percentage of each neuronal type in 3377 neurons sequenced is shown at the top of each bar. D) The number of mapped reads in each neuronal cell type. The fraction of reads mapped in each neuronal type accounts for all mapped reads in 3377 neurons is shown at the top of each bar. E) The fraction of the pCSM loci covering each cell type. F) The synthetic proportions of each neuronal cell type. The error bar shows the standard deviation of the synthetic proportions in 100 methylomes. Figure S3. Characteristics of pCSM loci identified from brain methylomes. A) A sketch map of pooling samples. B) Number of pCSM segments identified from neuronal and pooled methylome. “Vanished” represents the segments identified as pCSM segments within each neuronal cell population but identified as non-CSM segments in pooled sample. “Emerged” represents the segments identified as pCSM segments in pooled sample but identified as non-CSM segments within each individual cell population. “Derived” represents the segments identified as pCSM segments in both pooled sample and at least one neuronal cell population. C) Venn plot shows the overlap between pCSM segments identified from single-cell methylomes and those identified from the pooled methylome. D) The distribution of pCSM loci across various genomic features compared to those of control regions. [file 13072_2019_310_MOESM3_ESM.docx]

**Additional file**

**Virtual Methylome Dissection Facilitated by Single Cell Analyses**

**Contents:**

Tables S1–S3

Figures S1–S3


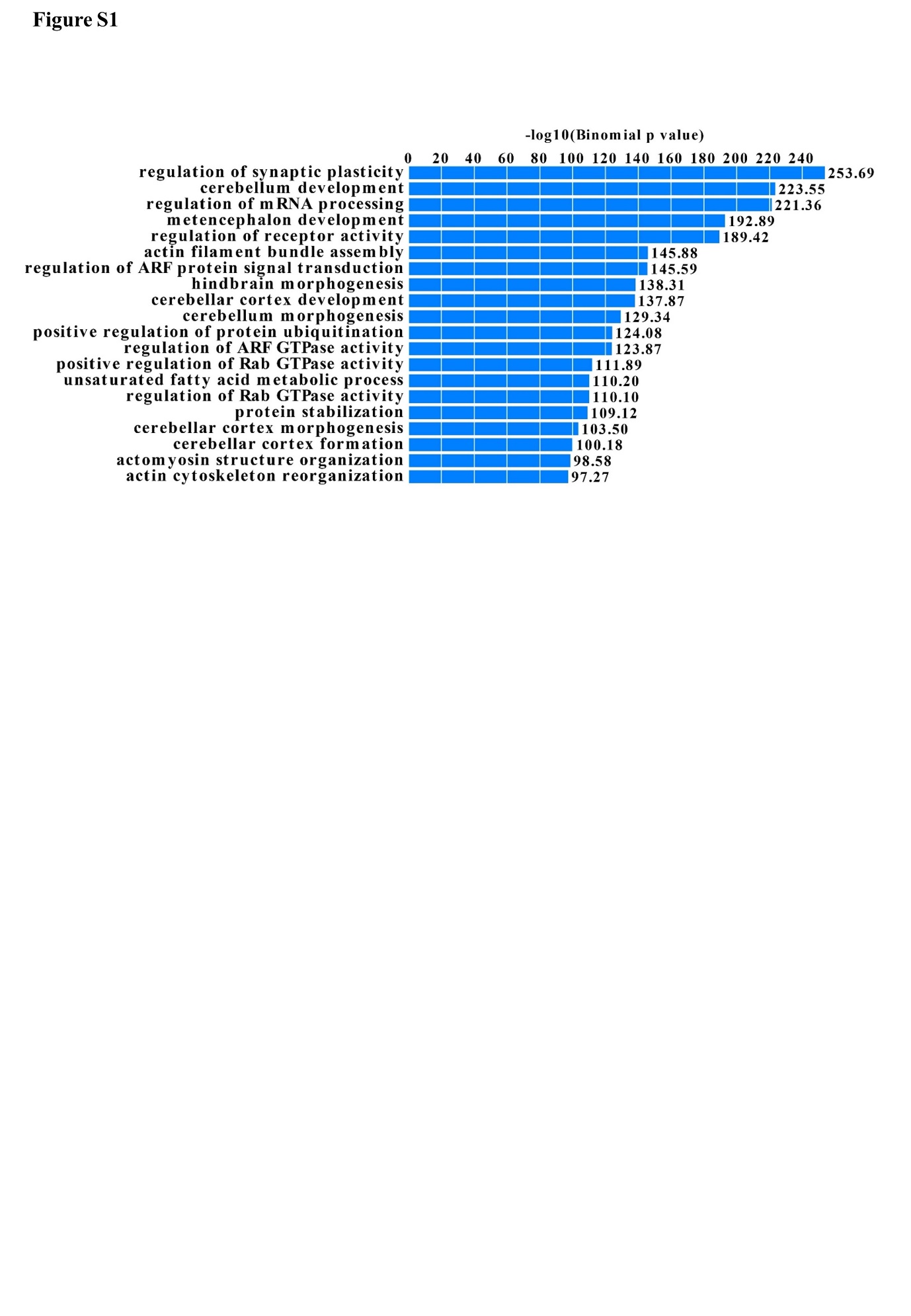


**Figure S1. Functional enrichment of genes with pCSM loci overlapped with enhancer or promoter histone marks.**

**
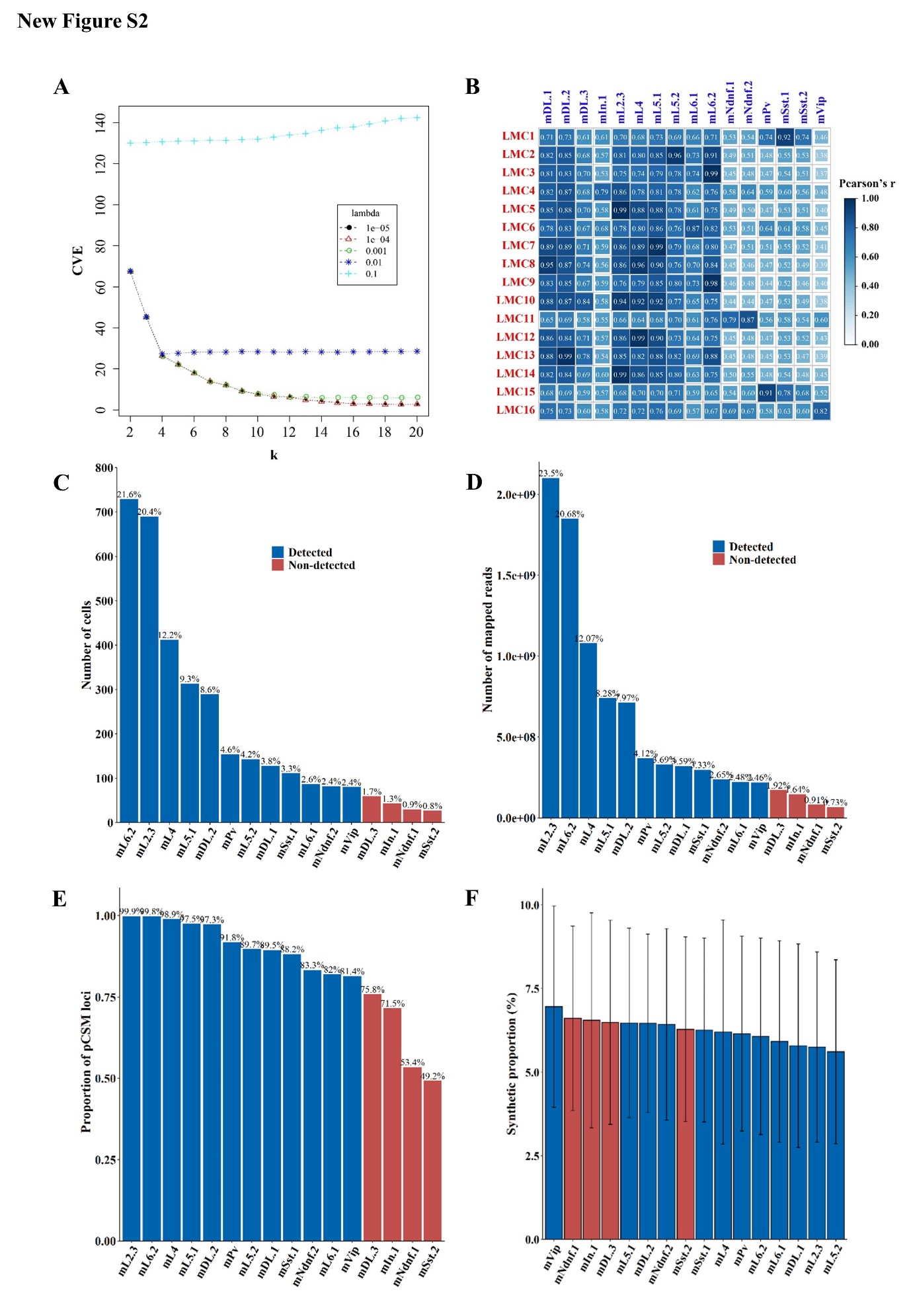
**

**Figure S2.** **Virtual methylome dissection using eigen-pCSM loci. A)** Selection of parameter λ by cross-validation. **B)** Pearson’s correlation coefficient between real cell types and NMF predicted cell types. **C)** The number of cells in each neuronal cell types identified by Luo et al. The percentage of each neuronal type in 3,377 neurons sequenced is shown at the top of each bar. **D)** The number of mapped reads in each neuronal cell type. The fraction of reads mapped in each neuronal type accounts for all mapped reads in 3,377 neurons is shown at the top of each bar. **E)** The fraction of the pCSM loci covering each cell type. **F)** The synthetic proportions of each neuronal cell type. The error bar shows the standard deviation of the synthetic proportions in 100 methylomes.


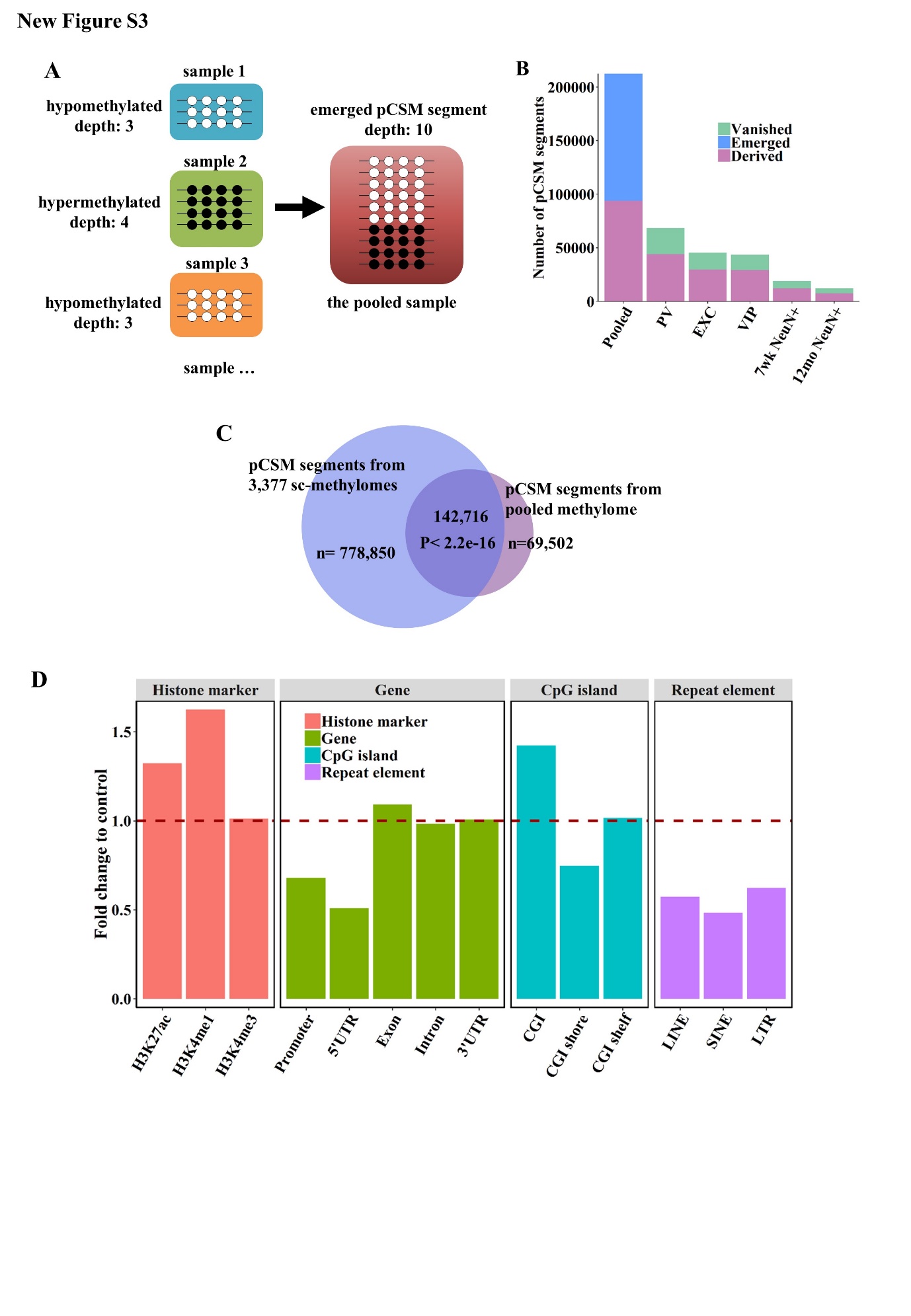


**Figure S3.** **Characteristics of pCSM loci identified from brain methylomes. A)** A sketch map of pooling samples. **B)** Number of pCSM segments identified from neuronal and pooled methylome. “Vanished” represents the segments identified as pCSM segments within each neuronal cell population but identified as non-CSM segments in pooled sample. “Emerged” represents the segments identified as pCSM segments in pooled sample but identified as non-CSM segments within each individual cell population. “Derived” represents the segments identified as pCSM segments in both pooled sample and at least one neuronal cell population. **C)** Venn plot shows the overlap between pCSM segments identified from single-cell methylomes and those identified from the pooled methylome. **D)** The distribution of pCSM loci across various genomic features compared to those of control regions.
